# Reduced erbium-doped ceria nanoparticles: one nano-host applicable for simultaneous optical down- and up-conversions

**DOI:** 10.1186/1556-276X-9-231

**Published:** 2014-05-13

**Authors:** Nader Shehata, Kathleen Meehan, Ibrahim Hassounah, Mantu Hudait, Nikhil Jain, Michael Clavel, Sarah Elhelw, Nabil Madi

**Affiliations:** 1 Bradley Department of Electrical and Computer Engineering, Virginia Tech, Blacksburg, VA, USA; 2 Department of Engineering Mathematics and Physics, Faculty of Engineering, Alexandria University, Alexandria, Egypt; 3 Institute for Critical Technology and Applied Science (ICTAS), Blacksburg, VA, USA; 4 Center of Advanced Materials (CAM), Qatar University, Doha, Qatar

**Keywords:** Ceria nanoparticles, Erbium dopant, Fluorescence, Up-conversion

## Abstract

This paper introduces a new synthesis procedure to form erbium-doped ceria nanoparticles (EDC NPs) that can act as an optical medium for both up-conversion and down-conversion in the same time. This synthesis process results qualitatively in a high concentration of Ce^3+^ ions required to obtain high fluorescence efficiency in the down-conversion process. Simultaneously, the synthesized nanoparticles contain the molecular energy levels of erbium that are required for up-conversion. Therefore, the synthesized EDC NPs can emit visible light when excited with either UV or IR photons. This opens new opportunities for applications where emission of light *via* both up- and down-conversions from a single nanomaterial is desired such as solar cells and bio-imaging.

## Background

Optical nanostructures that emit visible light when excited by ultraviolet (UV) or infrared (IR) photons have been extensively studied for applications that include bioimaging [1,2], solar energy [3,4], and optical gas sensors [5,6]. Research on one of these nanomaterials, cerium oxide (ceria) nanoparticles, has shown that its material properties are extremely well suited for a lot of applications; ceria can be employed as the optical active agent in UV absorbents and filters [7], gas sensors [8], and bioimaging media [9]. Visible emission from either UV excitation (down-conversion) or IR excitation (up-conversion) can be obtained from ceria nanoparticles. However, both up- and down-conversion processes involve different physiochemical properties in ceria and optimization of each optical process *via* various nanoparticle synthesis and post-growth procedures tends to quench the efficiency of the other process.

For example, ceria nanoparticles synthesized at or near room temperature by a chemical precipitation method will fluoresce in the visible wavelength region, *λ*_peak_ approximately 520 nm, when excited by near-UV photons (*λ*_exc_ approximately 430 nm) [10]. The down-conversion process requires that the cerium ions are in the Ce^3+^ state and are associated with oxygen vacancies, which implies that ceria nanoparticles contain Ce_2_O_3_ is a direct semiconductor [11]. To obtain visible light *via* up-conversion, ceria nanoparticles must be doped with certain lanthanides, such as erbium, then annealed at temperatures above 700°C [12]. Ceria is a low-phonon host for the erbium ions, which act as optical centers that convert the energy from absorbed IR photons into visible light [13]. However, the presence of the negative-association energy element, erbium, and the high temperature anneal causes the dominant ionization state of cerium ions to be in the Ce^4+^ state where Ce^4+^ ions bond with oxygen to form CeO_2_, an indirect semiconductor [10,14,15]. Hence, the down-conversion emission efficiency of the erbium-doped ceria nanoparticles (EDC NPs), particularly after the thermal anneal, is low [10]. On the other hand, there is no observable up-conversion emission from undoped ceria nanoparticles or from ceria nanoparticles doped with positive association energy lanthanide. Thus, to optimize the properties of ceria nanoparticles for the two optical conversion processes, it has been required two different nanoparticle synthesis and post-processing procedures.

**Figure 1:**
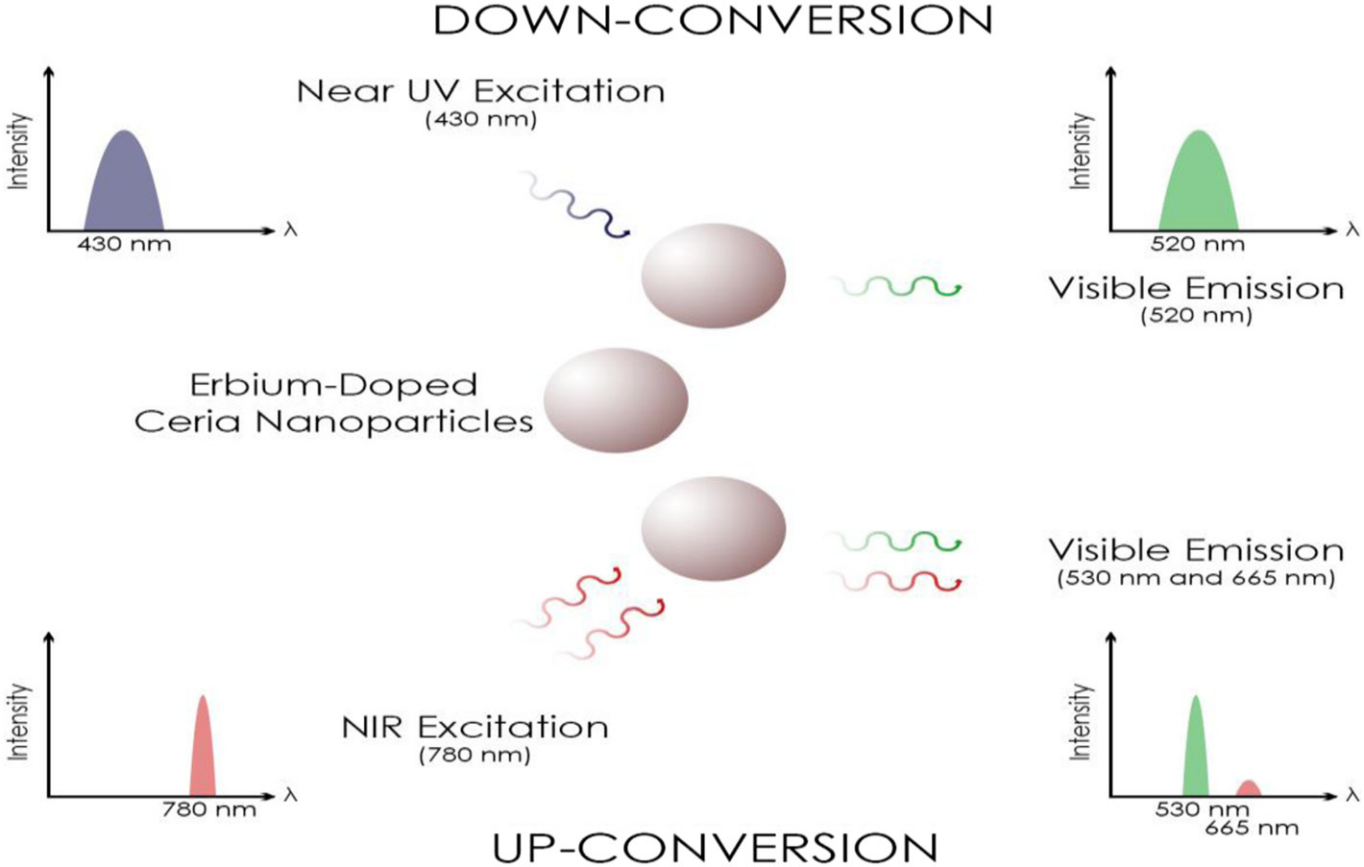
**Illustrative diagram demonstrating usage of EDC NPs in generating visible light.** Simultaneous UV (down-conversion) and IR (up-conversion) excitations.

As shown in the illustrative diagram of Figure 1, this work introduces a reduced EDC NPs that have the unique material properties to act as an optical medium for both down-conversion and up-conversion in the same time to generate multi-wavelength visible emissions under near UV and IR excitations, respectively. The used synthesis process results in a high concentration of Ce^3+^ ions associated with the oxygen vacancies in ceria, which is required to obtain high fluorescence efficiency in the down-conversion process. Simultaneously, the synthesized nanoparticles contain the molecular energy levels of erbium that are required for up-conversion. Therefore, the EDC NPs synthesized using this procedure can emit visible light when excited with either or both UV or IR photons. This work is the first, to the best of the authors' knowledge, to offer one optical nanomaterial for both up- and down-conversions simultaneously. This opens new opportunities for applications where emission of visible light *via* both up- and down-conversions from a single nanomaterial is desired.

## Methods

EDC NPs are prepared using the chemical precipitation technique which is relatively simple and inexpensive synthesis process [16,17]. Cerium (III) chloride (0.475 g) and erbium (III) chloride (0.025 g) are dissolved in de-ionized (DI) water (40 mL) to obtain a 5% weigh ratio of erbium to cerium in the synthesized nanoparticles. This weight ratio is selected after a study by the authors of EDC NPs, synthesized using the same process, in which it was found that optimal concentration of erbium in ceria for up-conversion is 5 wt.% which is close to the quenching ratio mentioned by another research group [13]. The solution is stirred constantly at 500 rpm in a water bath, while the temperature of the water bath is raised to 60°C, and ammonia (1.6 mL) is then added to the solution. The solution is kept at 60°C for 1.5 h and, then, the solution is stirred for another 22.5 h at room temperature. The colloidal solution is centrifuged and washed with DI water and ethanol to remove any unreacted cerium and ammonia. Then, the wet powder is dried on a hot plate. The thermal anneal of the dried nanoparticles is performed in a tube furnace (CM Furnace, Model 1730-20HT, Bloomfield, NJ, USA) with an atmosphere of hydrogen and nitrogen gases that are injected into the furnace at flow rates equal to 10 and 5 standard cubic feet per minute (scfm), respectively, for 2 h at temperatures of 700°C, 800°C, and 900°C. The gases during the anneal assist with the reduction of the cerium ions from the Ce^4+^ to Ce^3+^ ionization states and the creation of the oxygen vacancies [18], while the thermal energy available during the high temperature anneal promotes the formation of the molecular energy levels of erbium inside the ceria host [19].

**Figure 2:**
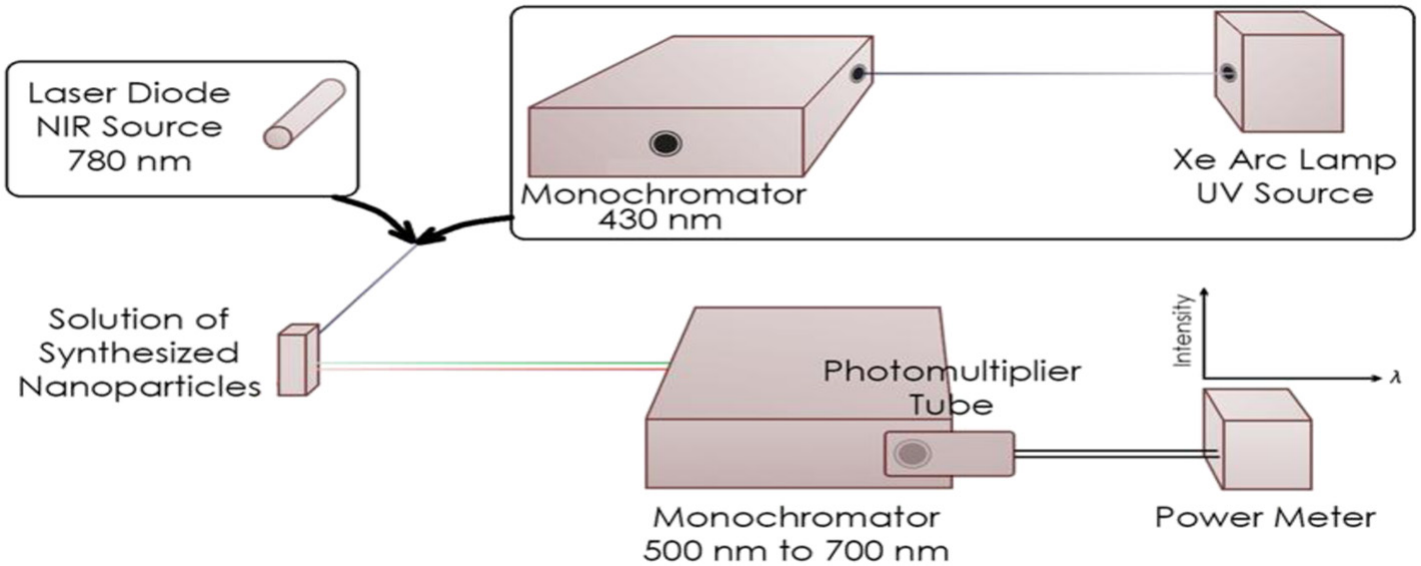
Experimental setup used to measure the down- and up- conversions.

The optical absorption is measured using a dual-beam UV-vis-NIR spectrometer (UV-3101PC Shimadzu, Kyoto, Japan). Using the data from the linear region of absorption spectrum, the allowed direct bandgap can be calculated using Equation 1 [20].

(1) $$\alpha \left(E\right)=A{\left(E-E_{g}\right)}^{1/2}$$

where *α* is the absorbance coefficient, *A* is a constant that depends on the effective masses of electrons and holes in the material, *E* is the energy of the absorbed photon, and *E*_*g*_ is the allowed direct bandgap. Following the annealing procedure, 0.02 mg of nanoparticles is re-suspended in 10 mL of DI water prior to optical characterization. The colloidal solution is illuminated with near-UV light in an experimental apparatus that was designed to measure the down-conversion process, as described in Figure 2. To measure the up-conversion emission when the samples are excited with near-IR photons, a 780-nm IR laser module is substituted for the UV lamp with the first monochromator and the remaining equipment in the experimental setup is unchanged. A transmission electron microscope (TEM), Phillips EM 420 (Amsterdam, The Netherlands), is used to image EDC NPs. The mean diameter of the nanoparticles is calculated using ImageJ software. The operating parameters of the XRD, a PANalytical's X'Pert PRO X-ray diffractometer (Almelo, The Netherlands), are 45 KV, 40 A, and CuKα radiation (*λ* = 0.15406 nm).

## Results and discussions

**Figure 3:**
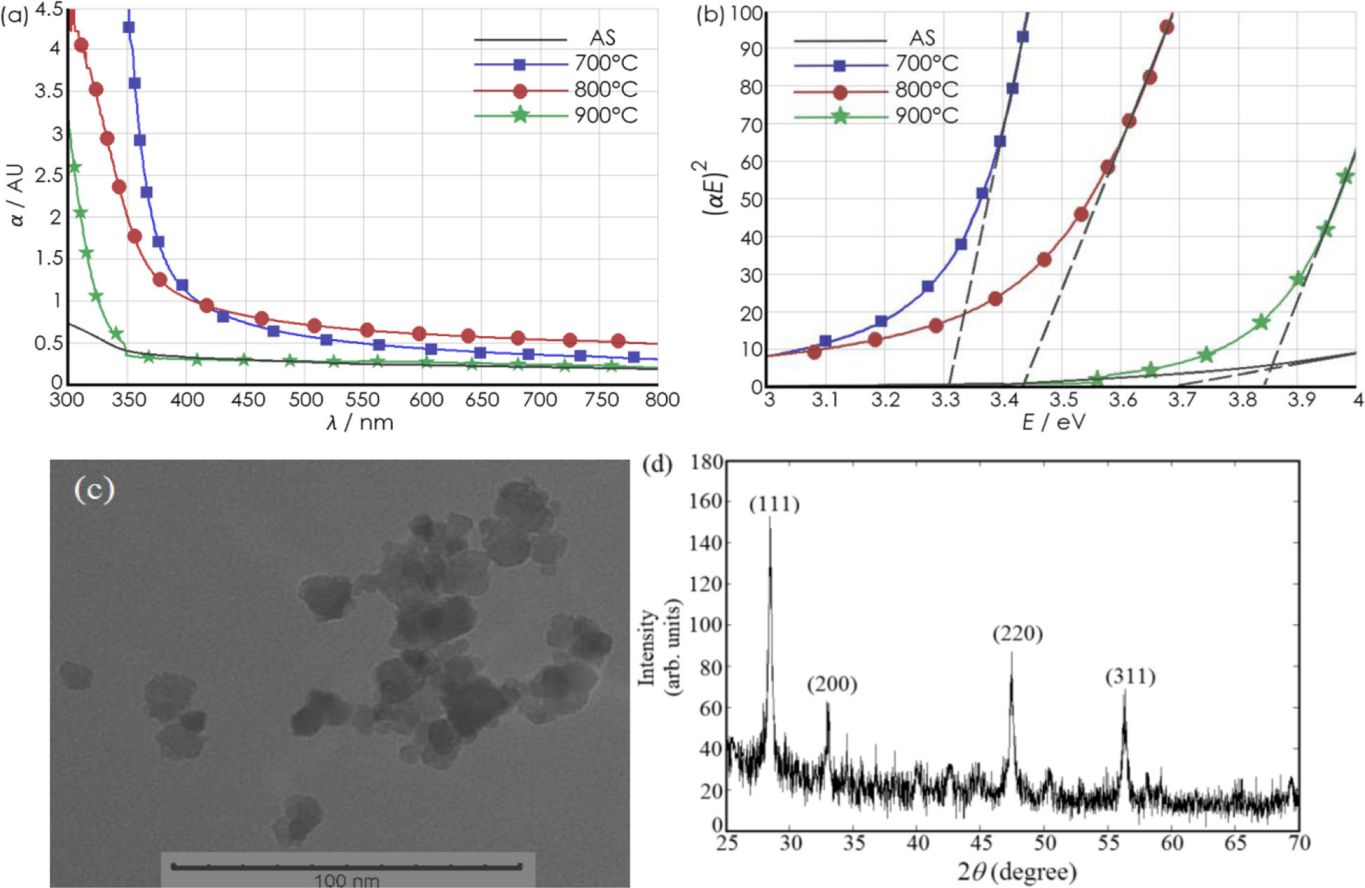
**Absorbance dispersion curves (a), graphs to calculate direct bandgap (b), SEM image (c), and XRD pattern. (a)** Absorbance dispersion curves for the EDC NPs annealed at 700°C, 800°C, and 900°C; **(b)** the graphs used to calculate the direct bandgap of the annealed EDC NPs, and **(c)** a SEM image of and **(d)** XRD pattern from a sample of the EDC NPs following the 800°C anneal, as a representative example (AS, as-synthesized or unannealed).

The optical absorption spectra of the synthesized EDC NPs are plotted in Figure 3a. The corresponding values for the calculated allowed direct bandgaps of the annealed samples are shown in Figure 3b. Compared to the non-annealed EDC NPs, it can be observed that the bandgap is biased towards 3 eV, which is approximately the bandgap energy for Ce_2_O_3_. Thus, there is a high concentration of Ce^3+^ and oxygen vacancies [10], after the anneal at 700°C. The bandgap energy of the EDC NPs is slightly larger following the 800°C anneal, indicative of a lower concentration of Ce^3+^ in the nanoparticles [21]. However, there is a significant shift in the bandgap of the EDC NPs annealed at 900°C, which suggests that the cerium ions in the EDC NPs have been almost completely converted from the Ce^3+^ ions into Ce^4+^ states during the 900°C anneal, similar to the unannealed composition.

The annealed EDC NPs are imaged using TEM and compared to that of the unannealed EDC NPs. A representative image is shown in Figure 3c; it is an image of the EDC NPs after an 800°C anneal. Following the anneal temperature range between 700°C to 900°C, the mean diameter is found to be in the range of 9 to 13 nm as compared to a mean diameter of 7 nm for the unannealed (as-synthesized) EDC NPs. The synthesized EDC NPs have mean diameter smaller than other optical nanoparticles that have been studied as an optical active medium for down- or up-conversion [22-25]. An X-ray diffraction (XRD) pattern is presented in Figure 3d, measured on a sample of the EDC NPs annealed at 800°C, to demonstrate that the predominant nanostructure of the EDC NPs is cerium dioxide [10,26]. The diffraction peaks in the XRD patterns measured on the as-synthesized EDC NPs and the nanoparticles annealed at 700°C and 900°C also are characteristics of ceria.

**Figure 4:**
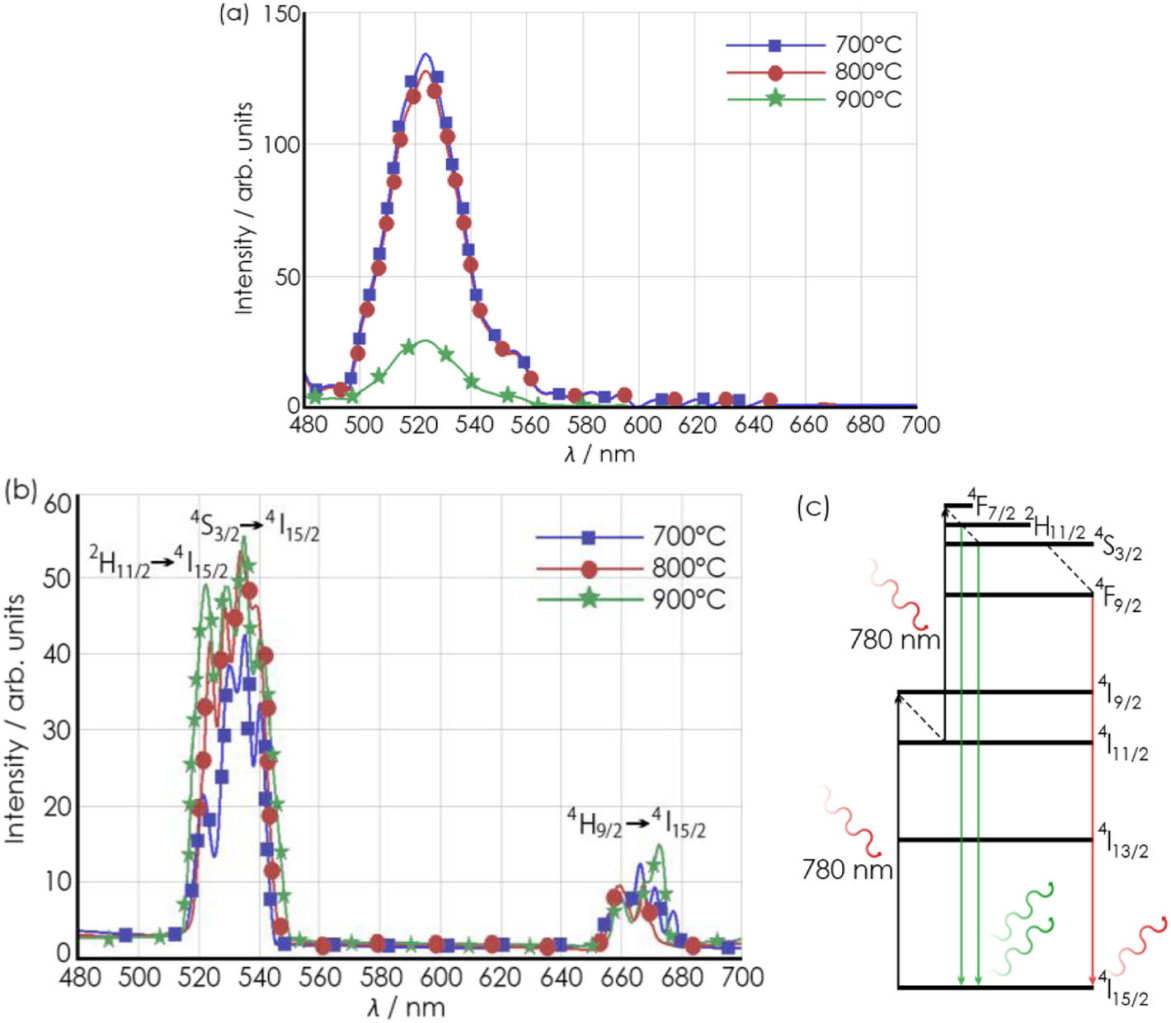
**Spectra of down-converted and up-converted emissions (a,b) and diagram of up-conversion energy mechanisms (c). (a)** When excited at 430 nm and **(b)** when excited at 780 nm measured on samples of EDC NPs annealed at 700°C, 800°C, and 900°C. Dotted lines in **(c)** are non-radiative transitions.

Under near-UV (*λ* = 430 nm) excitation, the visible emission from the EDC NPs is centered around 520 nm, as shown in Figure 4a. As can be seen, the anneal conditions at 700°C and 800°C are optimum for the down-conversion process, which involves the radiative relaxation of 5*d* to 4*f* transition of an excited Ce^3+^ ions in Ce_2_O_3_ that results in broadband emission in the green wavelength [10,27]. A further explanation of the down-conversion process is as follows: When the EDC NPs containing some fraction of Ce_2_O_3_ are illuminated with near-UV light, some fraction of the valence band electrons are excited to an oxygen vacancy defect state located within the CeO_2_ bandgap. From the defect state, the electron undergoes multiple transitions as it returns to the ground state. Only one of the transitions results in radiative emission and the other transitions are non-radiative. The rate of spontaneous emission from the EDC NPs, which is proportional to the amplitude of the peak intensity of the emitted fluorescence spectrum, is also proportional to the concentration of the oxygen vacancies that create the defect state; Ce^3+^ ions, near the conduction band. Therefore, the EDC NPs that have the strongest fluorescence, when annealed at 700°C, contain the highest concentration of Ce^3+^ states [10]. The peak amplitude of the down-conversion emission decreases with increasing anneal temperature, indicating that the higher temperature annealing reduce the concentration of oxygen vacancies and Ce^3+^ ionization states. This is most clearly observed in samples annealed at 900°C.

When the EDC NPs are excited by near-IR (*λ* = 780 nm) photons, visible emission is observed at two regions in the visible wavelength range; the primary emission is between 520 to 560 nm and a much smaller emission is found at 660 to 680 nm, as shown in Figure 4b. We hypothesize that erbium ions form stable complexes with oxygen in the ceria host during the anneal and the crystalline structure of the nanoparticle improves, both of which increase the efficiency of Er^+3^ ions to act as optically active centers for up-conversion [19]. The results include a slight improvement of the intensity of the up-conversion emission with increasing annealing temperature. A portion of the Dieke diagram is illustrated in Figure 4c, which shows that excited state absorption (ESA) is possible. First, the erbium ion is excited from ^4^I_15/2_ level to ^4^I_9/2_[13]. From the ^4^I_9/2_ state, the excited Er^+3^ ion non-radiatively relaxes to the ^4^I_11/2_ state. If a second 780-nm photon interacts with the excited Er^+3^ ion, an ESA process occurs, which excites the erbium ion to the level of ^4^ F_7/2_. After a series of non-radiative relaxations to lower levels such as ^2^H_11/2_, ^4^S_3/2_, and ^4^ F_9/2_, radiative relaxation to the ^4^I_15/2_ state occurs and visible emission results; green photons are emitted during the transitions from ^2^H_11/2_ and ^4^S_3/2_ to ^4^I_15/2_ while red photons are emitted during the ^4^ F_9/2_ to ^4^I_15/2_ transition.

## Conclusions

In conclusion, this paper presents a study on a new synthesized nanomaterial, EDC NPs, that emit photons in the visible wavelength range when illuminated by two different excitation sources: near-UV light (430 nm) and near-IR (780 nm) light. When the excitation source is near-UV light, a down-conversion process results in a broad emission peak centred at 520 nm. Up-conversion of the near-IR light is responsible for the narrower bands of green and red emission. Anneals at temperatures of 700°C and 800°C in a hydrogen-nitrogen atmosphere reduces the cerium ions from the Ce^4+^ to Ce^3+^ state. The reduced state (Ce^3+^) associated to oxygen vacancies form defect states that are responsible for the down-conversion emission. At the same time, the erbium ions form complexes with oxygen, which improves up-conversion efficiency. EDC NPs, with average diameter of 9 to 13 nm, may be employed in new applications in biomedicine, solar cell technology, and gas sensing, where an optical nanomaterial that can emit *via* either up- or down-conversion may be of value.

## Competing interests

The authors declare that they have no competing interests.

## Authors' contributions

NS carried out the nanoparticles synthesis, absorbance measurements, and up/down optical conversion setup design and measurements. KM guided NS in the overall work such as the synthesis procedure and fluorescence setup design in addition to the critical revision of the paper. IH and SE contributed critically in the synthesis of the reduced nanoparticles in addition to the manuscript writing. MH and NJ were responsible for XRD measurements and analysis. MC contributed in the nanoparticle synthesis and data collection. NM shared in synthesis procedure guidance and manuscript revision. All authors read and approved the final manuscript.
